# Nick Kitson MB, BS, FRCPsych: Formerly consultant psychiatrist, Cornwall Partnership NHS Foundation Trust and Springfield University Hospital, honorary senior lecturer, St George's Hospital Medical School

**DOI:** 10.1192/pb.bp.114.050245

**Published:** 2015-08

**Authors:** Richard Laugharne

**Figure:**
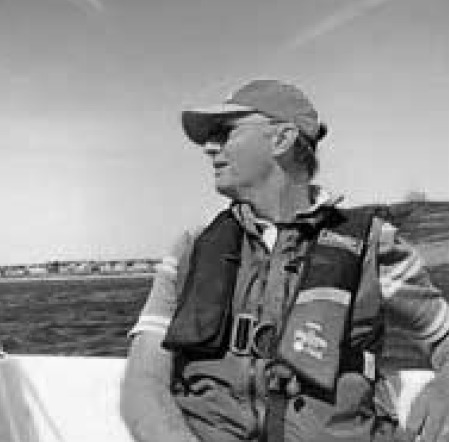


Nick Kitson, who died suddenly at the age of 64 while on holiday on 24 February 2014, was a pioneer and national expert in the psychiatric disorders of deaf people. He was the Founding Chair of the British Society for Mental Health and Deafness and the longest serving president of the European Society for Mental Health and Deafness (1990-1997), remaining honorary member and honorary vice president of each, respectively. He was clinical advisor to the Towards Equity and Access Implementation Panel (the Department of Health and National Institute for Mental Health England commissioning panel) in 2006/2007 and ‘responsible owner’ of a National Deaf Mental Health Commissioning Project. He remained a trustee of and medical advisor to the Sign health charity and awarded the title of Pioneer. He was joint editor of the standard introductory textbook *Mental Health and Deafness*. At the World Congress of Mental Health and Deafness held in 2014, a prize for the best poster was established in his name.

Nick completed his medical training at St Bartholomew's Hospital in 1975. He was appointed consultant psychiatrist at Springfield University Hospital and honorary senior lecturer at St George's Medical School in 1984. He was clinical director for the Specialist Mental Health Services for the Deaf Community at Springfield and medical director of Pathfinder NHS Trust from 1994 to 1998. When he became responsible for the psychiatric care of people with deafness, his health authority gave him 9 months to learn sign language. He visited psychiatric units for deaf people in America and realised the importance of employing deaf people fluent in sign language. At the time he wrote about sign language: ‘It is a very sophisticated language capable of expressing everything you can say in English.’ The service for deaf people that he led and developed for 18 years, with his wife Karen, covered the southern half and then a third of England. He became a Member of the Royal College of Psychiatrists in 1980 and was elected a Fellow in 1996.

While working as a consultant, he trained in short-term dynamic therapy in 1984 and group analytic psychotherapy in 1989. He became an associate member of the London Centre for Psychotherapy (British Psychotherapy Foundation) in 1989 and a Full Member in 1996, supervising short-term dynamic psychotherapy at the Tavistock and latterly at the London Centre for Psychotherapy. Psychotherapy was always an integral part of his clinical practice. He provided strong support for Jane Douglas, the first profoundly deaf person to train as a psychoanalytic psychotherapist.

Knowing the West Country well, Nick left London and moved to Cornwall in 2002. He initially worked as a community and in-patient psychiatrist covering the St Austell area but, wishing to reduce his workload owing to ill health, he became part-time consultant to the psychiatric intensive care unit (PICU). The service was awarded the National PICU of the Year in 2005. Formally retiring in 2009, he continued as his own locum for the PICU, regularly sailing and taking trips abroad with Karen and his family.

Nick was a hugely respected consultant psychiatrist. His colleagues frequently sought his advice in difficult circumstances, and the advice he gave was wise and supportive. He was down-to-earth and sensible; he never pretended he knew the answer when he did not, but his advice was even sounder in these circumstances. He continued to use his therapeutic skills after retirement and remained enthusiastic and determined to help the most challenging and difficult patients.

He will be sorely missed by his colleagues but his wise leadership will continue to be influential. He is survived by Karen, his two daughters and his grandchildren.

